# The Economic Impact of Exchanging Breeding Material: Assessing Winter Wheat Production in Germany

**DOI:** 10.3389/fpls.2020.601013

**Published:** 2020-12-23

**Authors:** Sophia Lüttringhaus, Christoph Gornott, Benjamin Wittkop, Steffen Noleppa, Hermann Lotze-Campen

**Affiliations:** ^1^Sustainable Land Use and Climate Change, Department of Agricultural Economics, Humboldt-Universität zu Berlin, Berlin, Germany; ^2^HFFA Research GmbH, Berlin, Germany; ^3^Potsdam Institute for Climate Impact Research, Member of the Leibniz Association, Potsdam, Germany; ^4^Agroecosystem Analysis and Modelling, Faculty of Organic Agricultural Sciences, University of Kassel, Kassel, Germany; ^5^Department of Plant Breeding, IFZ Research Centre for Biosystems, Land Use and Nutrition, Justus Liebig University Giessen, Giessen, Germany

**Keywords:** economic surplus analysis, plant breeding, breeder’s exemption, exchange of breeding material, winter wheat production, Germany, pedigree analysis, impact assessment

## Abstract

Climate change impacts imply that the stabilization and improvement of agricultural production systems using technological innovations has become vital. Improvements in plant breeding are integral to such innovations. In the context of German crop breeding programs, the economic impact of exchanging genetic material has yet to be determined. To this end, we analyze in this impact assessment the economic effects on German winter wheat production that are attributable to exchanging parental material amongst breeders in the breeding process. This exchange is supported by the breeders’ exemption, which is an integral part of the German plant variety protection legislation. It ensures that breeders can freely use licensed varieties created by other breeders for their own breeding activities and aims to speed up the development of improved varieties. For our analysis, we created a unique data set that combines variety-specific grain yield, adoption, and pedigree information of 133 winter wheat varieties. We determined the parental pedigree of each variety to see if a variety was created by interbreeding varieties that are internal or external to its specific breeder. Our study is the first that analyzes the economic impact of exchanging genetic material in German breeding programs. We found that more than 90 % of the tested varieties were bred with exchanged parental material, whereby the majority had two external parents. Also, these varieties were planted on an 8.5 times larger area than the varieties that were bred with two internal parents. Due to lower adoption, these only contributed 11 % to the overall winter wheat production in Germany, even though they yielded more. We used an economic surplus model to measure the benefits of exchanging parental breeding material on German winter wheat production. This resulted in an overall estimated economic surplus of 19.2 to 22.0 billion EUR from production year 1972 to 2018. This implies tremendous returns to using the breeder’s exemption, which, from an economic perspective, is almost cost-free for the breeder. We conclude that the exchange of breeding material contributes to improving Germany’s agricultural production and fosters the development of climate-resilient production systems and global food security.

## Introduction

Wheat (*Triticum aestivum*) as a crop is vital for global food security. On a global scale, it provides about 20% of dietary calories and proteins (Shiferaw et al., 2013). In 2017, Germany was the tenth largest wheat producer in the world (FAOSTAT, 2020), representing a high potential production area for wheat due to its favorable and temperate climate as well as good agronomic practices. The main wheat strain grown in Germany is winter wheat, which is grown on about 98% of all wheat areas in the country (BMEL, 2020). The crop is an important income source for German farmers as high-quality wheat is the most profitable among cereals and is the most important agricultural export product in Germany (BMEL, 2019).

Plant breeding has contributed considerably to the nutritional and economic importance of the crop, because modern cultivars provide higher yields and better quality as well as improved resistances and tolerances to abiotic and biotic stresses (Searchinger et al., 2014; Lantican et al., 2016; Voss-Fels et al., 2019). A large part of the innovation in breeding is claimed to be attributable to the vast pool of genetic resources that breeders can tap into to create new varieties (Hoisington et al., 1999).

Around the globe, the access to genetic resources is governed by various national legislations. In Germany plant variety protection is coupled with the breeder’s exemption that allows breeders to use released varieties created by other breeders to create new varieties in their breeding programs without costs. This creates an “open-source system” enabling breeders to access external varieties. Thanks to the breeder’s exemption, breeders can efficiently access a vaster pool of genetic resources that includes the newest varieties. The purpose of the breeder’s exemption is to enable and accelerate the breeding progress for the benefit of the society (UPOV, 1991) as well as broaden and actively conserve (*in situ*) plant genetic material. Furthermore, it is designed to enable a faster supply of high-yielding and sturdy varieties (UPOV, 1991) that can improve the income of farmers and other stakeholders along the wheat value chain (Lotze-Campen et al., 2015; Lantican et al., 2016). In systems without the breeder’s exemption, this access could be restricted by the owner of certain material and/or more costly (Byerlee and Dubin, 2009). A criticism of the breeder’s exemption is that other breeders can freely use innovations of other breeders and hence could reduce the marketability of the original variety by improving it (Heald and Chapman, 2012).

In the scientific literature there is a debate on the positive and negative effects of specific regulatory systems for plant variety protection. Heald and Chapman (2012) state that neither the diversity loss theory, i.e., that patent systems contribute to a loss in intra- and inter-specific diversity, nor the incentive-to-invent theory, i.e., that patent systems create a greater and faster stream of innovations, are supported by their data when analyzing the impacts of patent systems on crop diversity. Eaton (2002) claims that a reduced access to plant genetic material can trigger market concentration, prohibitive prices, and decrease diversity. To the best of our knowledge, the economic impact of sharing genetic material on the overall production of winter wheat in Germany or another world region has not been quantitatively analyzed so far.

This paper seeks to provide insights to this debate. We explore to what extent the sharing of genetic material that is enabled by the breeder’s exemption has contributed to an increased breeding success. In this assessment, we analyze if interbreeding with external parent material created benefits for winter wheat production in Germany. This research question is answered by creating a novel data set that combines variety-specific pedigree information with breeding-induced improvements. Here, we focus on the effect on grain yield, as this agronomic trait is very important for overall wheat production.

In this article, we present the used data sets in the first step (section “Data”). In the next step we describe the methodology, where we first describe how we derive the pedigree information and group the varieties according to their share of external parent material (sub-section “Pedigree Analysis and Grouping”). Then we explain the variables that are created to derive the economic surplus of sharing parent material (sub-section “Variable Creation and Group Comparison”) and lastly present the scenarios for the assessment. Subsequently, the results are presented (section “Results”) and discussed (section “Discussion”). The article concludes with final remarks (section “Conclusion”).

## Data

For our analysis we use three main data sets. The first one is a field trial data set on the phenotypic trait grain yield of winter wheat cultivars. The research consortium *Breeding Innovations in Wheat for Resilient Cropping Systems* (BRIWECS) generated the data set by multi-location and multi-year field trials with 191 winter wheat cultivars that were registered in Germany between 1966 and 2013. Varieties that were bred in the German Democratic Republic (GDR) are not considered in our analysis, as they were bred under a different system of plant variety protection and breeding as well as the overall economy was centrally regulated (Brandl, 2018). Main field trials were conducted in six locations during the growing seasons 2014–2015 and 2015–2016, with at least two replications and three different intensity levels of nitrogen fertilization and plant protection (for a detailed description of the trials and the data analysis see Voss-Fels et al., 2019). The goal of the trials was to measure the genetic improvement induced by breeding over time. For our analysis, we use the observational data of the variable grain yield under the high input treatment level that represents the standard input use under intensive wheat production conditions in Germany (applying 220 kg N ha^–1^ and the full recommended intensity of fungicides, insecticides and growth regulators).

The second data set contains the variety-specific multiplication area for seed production of German winter wheat varieties from 1971 to 2017. It was compiled by Kempf (2019) and originated from the publicly available data published in the German Federal Plant Variety Office’s descriptive variety lists (Federal Plant Variety Office [FPVO], 2017). It serves to approximate the variety-specific area planted as also conducted in similar studies such as Lotze-Campen et al. (2015). The multiplication area can also be employed to compare the market importance of varieties, i.e., which variety was reproduced and hence planted more or less than others. The extent of the multiplication area on which a variety is reproduced in 1 year provides insights into the extent to which farmers plant a specific variety the next year. Also, no comprehensive data is available on the variety-specific planted area.

The third data set contains the variety-specific pedigree information, i.e., the names of the breeders that bred the maternal and paternal varieties. Section “Pedigree Analysis and Grouping” explains how we generated it.

## Methods: Disentangling the Economic Impact of Exchanging Genetic Resources

To quantify the economic benefits to German winter wheat production that are attributable to the exchange of breeding material, we conduct several steps. First, we pursue a pedigree analysis for all varieties within the data set by grouping them according to the extent of genetic material exchange that was used to create them (see step 3.1). Then we establish four variables to compare the groups (3.2). We also define two scenarios to simulate German winter wheat breeding with different extents of material exchange and additionally create an economic surplus to monetize these scenarios (3.3).

### Pedigree Analysis and Grouping

The purpose of the first step is to determine to what extent breeders exchanged genetic resources when breeding each variety and accordingly group these varieties. For each variety, we determined the parents and their respective breeders. Here, three steps were necessary. First, we generated a pedigree data set with information from the Genetic Resources Information System for Wheat and Triticale (GRIS) (Martynov et al., 2017), which is maintained by the International Maize and Wheat Improvement Center (CIMMYT) and the N. I. Vavilov Research Institute of Plant Industry^1^. Second, this data set was validated with pedigree information from the Federal Association of German Plant Breeders (Bundesverband deutscher Pflanzenzüchter). In that manner, we were able to generate 133 comparable entries with enough pedigree information. These make up 70% of the cultivars planted in the field trials.

Third, we checked, if a variety’s breeder interbred with external material from other breeders to create that specific variety. To this end, we compared each variety’s breeder name with those of the two parental breeders. When the names were similar, the parental line was considered as an internal variety. If this was not the case, we labeled the parent as external. Parental lines created via multiple crosses with at least one external variety were also labeled as external, even though the breeder of a variety produced the cross. We followed this approach as it provides a clear cut between internal and external parental material, which is fundamental for our analysis. We are aware that especially during the 1990s many German breeding companies were consolidated and hence along with the breeders, breeding programs, wheat crosses and knowledge were assimilated into other companies. On this account we assume that the material of a breeder that was later integrated into another company, is external to that company when the interbreeding took place after the consolidation.

Consequently, all varieties are assigned to three groups according to the extent of exchange in parent materials that took place to create a new variety:

• **Group 2E** comprises all varieties for which the breeder used two external parents. Both, mother, and father are from another breeder,
• **Group 1E** means that one parental line is from another breeder than the progeny. Hence, father or mother material was exchanged with other breeders,
• **Group E** combines both groups defined above and hence represents both groups whose varieties were created with exchanged parent material during the breeding process,
• **Group 0E** includes all varieties where breeders did not exchange parental material for the variety creation. Hence, the same breeder developed both, the parents as well as the progeny.

### Variable Creation and Group Comparison

In this step, we define the variables that are necessary to compare the groups: grain yield, multiplication area, weighted grain yield and group production.

The group comparison is validated with the non-parametric pairwise Wilcoxon rank sum test, which best suits the number of data points and does not assume known distributions.

#### Grain Yield

The phenotypic trait grain yield (dt ha^–1^) is available from the field trial data set. We concentrate on this trait because it is considered the priority of breeders, as it is crucial for a varieties’ market success, and also a major determinant for overall production levels, food security and farmers’ income.

#### Multiplication Area

The variety-specific multiplication area (in ha) is available from 1971 to 2017 for varieties bred in the Federal Republic of Germany and later in reunified Germany (Kempf, 2019). As we are interested in the extent of farmers’ adoption among varieties, the multiplication area serves as a proxy for the extent to which farmers planted certain varieties (farmers’ adoption). Like the variety-specific planted area, the multiplication area in year *t* reflects the area share planted with a certain variety in year *t+1*, only at another scale.

About 50% of winter wheat seeds in Germany are farmer-saved (Kate and Laird, 2020), i.e., farmers replant seeds from a specific wheat variety that they have produced themselves in a previous year. We assume that the share of farmer-saved seeds is equal across all varieties.

#### Weighted Grain Yield

With this variable we weigh the grain yield of each variety with its economic on-field importance. This gives more weight to those varieties with higher market penetration or multiplication area. Therefore, we create the variable weighted grain yield (*WY*) that weighs the yield by the respective variety-specific multiplication area and thus delivers a group yield average that considers varieties’ adoption and yield within a group.

For this purpose, we take the observations on grain yield for each variety *v* (*Y*_*v*_) and then multiply it with the respective multiplication area *M*_*v,t–1*_ in multiplication year *t*−1 (1971 – 2017), to account for the 1-year time lag between multiplication and production. This creates a timeline and is done for every group *g* (2E, 1E, 0E). To derive the average weighted yield of a group in production year *t* (*WY*_*g,t*_), we then divide the numerator by the sum of all varieties’ multiplication area that belong to one group:

(1) $$W\,Y_{g,t}=\frac{\sum_{v=1}^{V}\left(M_{v,t-1}\cdot Y_{v}\right)}{\sum_{v=1}^{V}M_{v,t-1}}$$

With
*WY*_*g,t*_  - Average weighted yield (dt ha^–1^) of each group *g*(*g* = 1…*G*), i.e., 2E, 1E, 0E, and each production year *t*,
*M*_*v,t–1*_  - Multiplication area (ha) of variety *v* in multiplication year *t-1* (1 year prior to the production year *t*),
*Y*_*v*_  - Average grain yield (dt ha^–1^) of each variety *v*(*v* = 1,…*V*), whereby *v* ∈ g.

In that manner, we compare all varieties that competed for market success in 1 year. This method reflects the preferences of farmers and considers that at the beginning of our time series less varieties were on the market.

#### Group Production

The group production (*Q*) calculates how much production each group contributes each year to the overall winter wheat production in Germany as given by EUROSTAT (2019, 2020). To this end, we divide each group’s production in a particular production year by the production of all groups, and then relate this share to the overall produced winter wheat quantity that year.

(2) $$Q_{g,t}=\frac{W\,Y_{g,t}\cdot M_{g,t-1}}{\sum_{g=1}^{G}W\,Y_{g,t}\cdot M_{g,t-1}}\cdot Q_{G\,e\,r,t}$$

With
*Q*_*g,t*_  - Yearly group production (million t) produced by the varieties of each group *g* given the total winter wheat production quantity in Germany in production year *t*,
*M*_*g,t–1*_  - Sum of the multiplication area (ha) for all varieties that belong to one group g and in multiplication year *t-1*,
*Q*_*Ger,t*_  - Winter wheat production quantity (million t) in Germany in year *t*.

### Scenario Definition and Economic Surplus Model

The goal of our analysis is to calculate whether the exchange of breeding material – or in other words the use of external parent material – generates an economic benefit in the German winter wheat production system. The use of protected varieties by breeders, hence exchanging genetic resources, is enabled by the breeder’s exemption, which is one pillar of the German and European plant variety protection. The German winter wheat breeding system is unique due to that regime, its market structure (mainly constituted of small and medium private enterprises), climatic conditions and demand requirements. As national seed markets and accompanying regulatory frameworks are highly diverse around the globe, it is difficult to compare the German system to a similarly structured seed sector and agriculture system without the exemption. Hence, we pursue a pairwise comparison of the four groups defined in sub-section “Pedigree Analysis and Grouping.” In Germany, breeders occasionally have bilateral agreements to access varieties and crosses of other breeders before they are officially released to accelerate the long breeding process. We assume that the existence of these agreements has no impact on the overall result, as all breeders have the possibility to establish contracts and most breeders are small- and medium-sized enterprises.

For the assessment, we define two scenarios to monetize the impact of exchanging parental breeding material on winter wheat production in Germany. Both scenarios describe *ex post* how the system would have differed with a limited exchange between breeders.

**Scenario I:** In this scenario we compare the varieties of group 2E, hence those with the maximum amount of parental exchange, with those of group 0E, which have no external parent.

**Scenario II:** Due to limited implementation of the breeder’s exemption, we assume that the exchange of parental material was prevented. Therefore, no varieties of groups 2E and 1E were planted in Germany, as these two groups are only possible when breeders can exchange material. We will use the joint group E to analyze this scenario, where German winter wheat production entirely depends on 0E-varieties that require no external parental breeding material.

#### Group Production According to Scenarios

##### Scenario I

To calculate how things would have changed if no 2E-varieties but instead 0E-varieties had been planted, we calculate the same as in equation (2), but substitute in the numerator the weighted yield of group 2E with the weighted yield of group 0E. The production quantity of scenario I *Q*_*S1,t*_ defined in equation (3), quantifies how much 0E-varieties would have produced if they had been planted on the area that was assigned to 2E-varieties.

(3) $$Q_{S\,1,t}=\frac{W\,Y_{0\,E,t}\cdot M_{2\,E,t-1}}{\sum_{g=1}^{G}W\,Y_{g,t}\cdot M_{g,t-1}}\cdot Q_{G\,e\,r,t}$$

##### Scenario II

Accordingly, this is done for scenario II, were we calculate what would have been produced with 0E-varieties on the area of group E (*Q*_*S2,t*_), which combines 2E and 1E.

According to these scenarios the production of a certain group can be zero in a certain production year if no varieties of that group has been multiplied in a previous year.

#### Economic Surplus Model

The benefits to German winter wheat production attributed to the exchange of parental material are estimated with an economic surplus model. Such a surplus model allows us to translate physical yield differences between the *status quo* and scenarios into monetary terms. Also, other similar studies such as Lantican et al. (2016) and Bernal-Galeano et al. (2020) took this approach to monetize the impact of specific varieties or contributions to crop pedigrees. The economic surplus quantifies the value of additional production that is attributable to exchanging parental material in the breeding process. To derive the time series of economic surpluses for scenario I *ES*_*S1,t*_, the annual production shifts (*Q*_2*E*,*t*_−*Q*_*S*1,*t*_) are multiplied with corresponding prices *p*_*t*_ for each production year:

(4) $$E\,S_{S\,1,t}=\left(Q_{2\,E,t}-Q_{S\,1,t}\right)\cdot p_{t}$$

The same is done for scenario II:

(5) $$E\,S_{S\,2,t}=\left(Q_{E,t}-Q_{S\,2,t}\right)\cdot p_{t}$$

The time series for the winter wheat prices is derived by matching the producer price index for bread wheat running from 1972 to 2018 (Destatis, 2019) with the reference wheat price from FAOSTAT (2020). Using this data, we receive annual economic surpluses in real prices.

## Results

### Extent of Parent Material Exchange and Use of the Breeder’s Exemption

After grouping the varieties, the descriptive analysis shows that most varieties were bred by exchanging genetic material with other breeders (see Table 1). Together, groups 2E and 1E make up more than 90% of the data set. Group 2E constitutes 61% (*n* = 81 varieties) and hence most of the analyzed German winter wheat varieties belong to this group. Group 0E contains only 12 varieties (9%).

**TABLE 1:** Distribution of varieties per group.

Group	Number of varieties per group	Percentage
2E	81	61%
1E	40	30%
0E	12	9%
Total	133	100%

*Source: Authors’ calculations.*

### Differences Between Groups

In the following we present the performance of each group considering the variables defined above.

#### Grain Yield

The descriptive analysis of the three groups shows that the unweighted grain yield is highest in group 0E (see Table 2). The Wilcoxon rank sum test shows that at α = 0.01, the difference in means between all groups are significantly different from 0 (see Annex Tables 1–4 for the *p*-values of the test for all variables). In addition to this result, a time bias can be noted with respect to the average yield for 0E-varieties. 0E-varieties were not available for multiplication in the years 1971–72, 74 and 83–90. Since a variety’s year of release considerably explains its yield level due to the breeding progress, the average for 0E-varieties in Table 2 is larger. This is because the value for 0E-varieties includes less varieties that were released in the beginning of our observation period than the values for 1E- and 2E-varieties. This effect is covered by the variable weighted grain yield.

**TABLE 2:** Overview of groups’ yearly averages per variable from production year 1972 to 2018.

Variable	2E	1E	E (2E and 1E)	0E
Grain yield (dt ha^–1^)	80.5	81.2	80.8	82.3
Multiplication area (ha)	218,622.4	117,170.6	335,792.9	32,618.0
Weighted yield (dt ha^–1^)	80.2	76.3	79.1	60.7
Group production (million t)	9.1	5.3	14.5	1.7

#### Multiplication Area

On average, group 2E was more multiplied than group 1E and 0E. Figure 1 shows the development of the multiplication area per group over time. It strikes that for eleven years (1971–72, 74, 83–90) no 0E-variety of our data set was multiplied After a small peak in the mid 1970s, only in 1991 0E-varieties are multiplied again.

**FIGURE 1:**
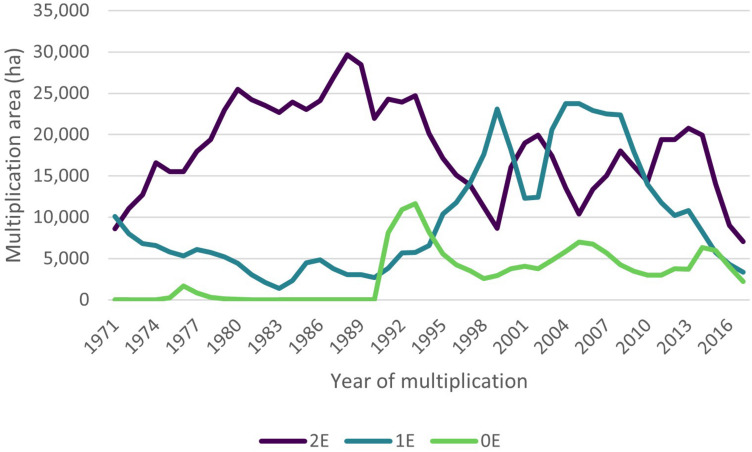
Development of multiplication area per group over time. Source: Authors’ calculations.

#### Weighted Grain Yield

When looking at Table 2, it strikes that the group ranking for weighted yield is reverse compared to the results for the variable (unweighted) grain yield. This is because on average the 0E-varieties were less multiplied than the other group’s varieties and hence had less market penetration. In some years even no 0E-varieties of our dataset were multiplied. The weighted yield is highest for group 2E, followed by group 1E and 0E.

**FIGURE 2:**
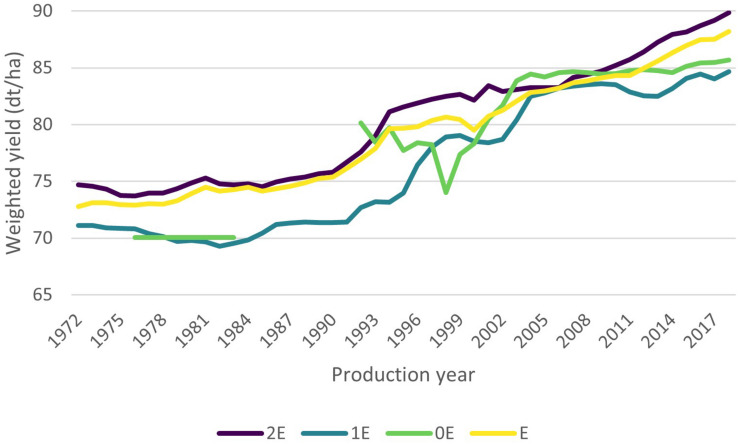
Development of the weighted yield over time. For a better readability of the graph, the line of group 0E is not represented when there were no 0E-varieties that were planted according to our data set. Source: Authors’ calculations.

Figure 2 shows the development of the weighted yield over the years. It becomes apparent that the weighted yield of all groups has a positive upward trend. In mid-2000, the differences between groups diminishes up until 2010 were it spreads out again. In some years, the weighted yield of group 0E exceeds that of the other groups while in other years no 0E-varieties of our data set have been multiplied.

#### Group Production

The group production quantities are identically ranked to the results of the multiplication area. According to our calculations, 2E-varieties have produced the largest share of overall German winter wheat production (57%), followed by group 1E (33%) and then group 0E (11%).

### Production Shifts and Economic Surplus

In both scenarios, 1E-varieties and the combined group of E-varieties created a positive production shift and also a positive economic surplus (see equation (4) and (5)) that is attributed to the exchange of parental breeding in the winter wheat breeding process. Over the period, the cumulative physical production shift of exchanging material amounts to 102.6 (scenario I) and 116.3 (scenario II) million t (see Annex Table 4). These quantities represent about 15% of the German winter wheat production in the given period.

When monetized with the economic surplus model, these physical production shifts amount to 19.2 billion EUR in scenario I and accumulated over the whole time from production year 1972 to 2018. On average a yearly surplus of 0.4 billion EUR was achieved. For scenario II, the sum of the economic surplus over the whole period is 21.9 billion EUR, while the yearly average totals 0.5 billion EUR.

**FIGURE 3:**
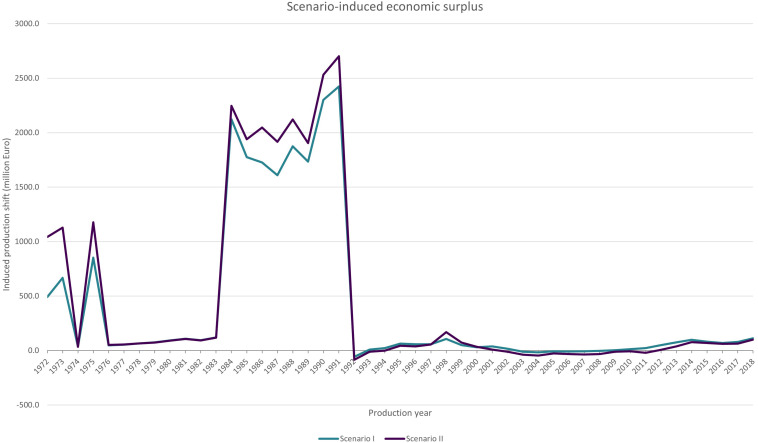
Economic surplus attributed to exchanging parental material in the winter wheat breeding process. Source: Authors’ calculations.

The economic surplus from production year 1972 to 2018 is displayed in Figure 3, the physical production shift follows the same curve progression. It clearly shows that the observed use of external parental breeding material, enabled by an unlimited and free exchange that is realized by the breeder’s exemption, created an economic surplus. Figure 3 shows that for most years the economic surpluses were small in comparison to the surges of specific years: In most years the surplus of scenario I ranged between 3.2 and 119.4 million EUR and between 6.5 and 169.0 million EUR for scenario II. In some years, the use of 2E- and E-varieties produced a small negative economic surplus. For scenario I it was negative in 1992 and from 2003 to 2008. The average economic surplus in these years was −15.9 million EUR. Due to the lower performance and adoption of 1E-varieties in comparison with 2E-varieties, for scenario II the surplus is negative from 1992 to 1994 and 2002 to 2011, and averages −27.6 million EUR. The surges in the economic surplus occur, when 2E- and 1E-varieties were adopted on larger areas than 0E-varieties. We can see a small surge from 1972 to 1975 and a large one from 1984 to 1991. The surges occurred after years when no 0E-varieties were multiplied. This means, that the group production of 0E-varieties was zero during these years.

The outcome of both scenarios is quite similar as 2E-varieties created the largest shifts within group E. We chose these two scenarios to show the effect of using varieties that were created with the “double” use of the breeder’s exemption (2E) with those of the combined group E that describes all varieties that were created with exchanged parental material.

## Discussion

To the best of our knowledge this is the first research paper that analyzes the extent to which external parental breeding material is used in German winter wheat breeding and the attributed economic surplus. We created a unique and novel data set that combines, for the first time, winter wheat varieties’ pedigree information with their yield trait and individual market penetration. Our pedigree analysis revealed that more than 90% of all German winter wheat varieties that were planted between 1972 and 2018 have at least one external parent. Hence, the work of breeders and a large part of the German wheat production relies on the genetic exchange enabled by the breeder’s exemption. Between 1972 and 2018, an economic surplus was generated that accumulates to 19.2 to 22.0 billion Euros. According to our analysis this surplus was driven by the large share of varieties bred with external parents within Germany’s portfolio of winter wheat varieties, farmers’ greater adoption compared to those without external parents, and relatively high wheat prices in the 1980s when much more varieties were planted that were bred with external parental material.

The breeder’s exemption provides full and free access to those varieties that are released. Louwaars(2019, p. 1), describes that the breeder’s exemption is an example for the “open innovation character of the plant breeder’s rights system” that prevails in Germany. Without that exemption breeders would need bilateral cooperation agreements or had to operate in regimes with patented varieties or variety traits. This would entail higher transaction costs and increase varietal development costs (Evenson and Santaniello, 2006; Louwaars, 2019), reduce the freedom to operate (Byerlee and Dubin, 2009) and might also increase breeding times. Owners of certain material could prohibit its use for other breeders and concentration processes could follow to create larger internal gene pools (Eaton, 2002; Brandl, 2018). Our data analysis shows that it is common for breeders to use external parents. In this manner, the genetic variation of crosses is enlarged, and the variability of outcomes increases. Matching two external parents or combining it with internal material helps to introduce genetically diverse material, hence creating varieties that are superior to their predecessors. Another reason for the large use of external material might be the fact that prior to the first crosses, breeders have more knowledge about the positive traits of an external variety than of less desired traits, as they have for their own breeding germplasm.

The results (see Table 2) show that group 0E has a yield advantage when comparing the unweighted grain yield. The yield performance ranking turns around when looking at the results for weighted yield, because on average varieties that were bred by exchanging parental material (groups 2E and 1E) were adopted on larger areas. But why did multipliers and farmers choose lower yielding varieties over higher yielding varieties? At first glance this might seem irrational, but farmers choose varieties based on their specific set of traits, which includes grain yield, but also resistances and tolerances to biotic and abiotic stresses, quality group, agronomic traits, previous experiences with the variety and personal preferences. Farmers’ choices are also influenced by seed availability which depends on the seeds offered by retailers and local agricultural cooperatives, and the recommendations by consulting businesses. Nevertheless, farmers’ preferences are reflected in their buying decisions (in particular in the longer-term perspective) and hence affect breeders and their future breeding strategies. Also, there is a high competition amongst German winter wheat breeders as the market consists of many small and medium, and also large enterprises. So, we can speculate that farmers adopted 2E- and 1E-varieties based on their unique set of traits which were conducive for the specific production conditions and purposes. Nevertheless, yield can be seen as a general performance indicator as other traits such as drought tolerance are mirrored in it. When a variety does not perform well under drought stress its yield will also reduce. Also, breeders’ market power can influence adoption. But the 0E-varieties in our data set were released by a very heterogenous group of small to large sized breeding companies. Hence one cannot attribute the higher adoption rate by the breeders’ market power or advantages regarding multiplication areas and marketing conditions. Moreover, due to fact that 0E-varieties in our data set were more common in years of registration later than 1990, it could be presumed that their yield advantage is due to the attested breeding progress (Voss-Fels et al., 2019). The varieties belonging to the groups 2E and 1E cover a longer registration period, hence the average of their unweighted grain yields was influenced by varieties released in the 1960s and 1970s with lower yield potentials. In our view, the most important reason for the superior unweighted grain yield performance of 0E-varieties is that two very successful varieties are within this group. These two varieties were largely adopted amongst German winter wheat farmers. Also, the other varieties in the group might have performed better under our trial environment (2015–2018) then under the environment following their release year. In that time after a variety’s release, farmers might have not preferred them due to disease susceptibility.

This adoption advantages can be scaled up to the overall winter wheat production in Germany. The group production shows that the use of 2E- and 1E-varieties has produced the largest share of German winter wheat production since the 1970s. Again, this can be explained by a larger adoption rate of 2E- and 1E-varieties and by the fact that more than 90% of all winter wheat varieties in our data set belong to these groups. Also, the economic surplus and thus the monetized impact of using varieties with external parent material is large when using weighted grain yield as an indicator.

We use the multiplication area to include variety adoption of farmers in our analysis. The multiplication is the only variety-specific and long-term data available to measure adoption of winter wheat varieties. On average our data set with the complete pedigree makes up 66% of the overall German multiplication area used for winter wheat (see Annex Figure 1).

A direct comparison of our results with the literature requires broadening the research question, looking at more geographical areas and including the economic impact of general agricultural research and development (R&D). There are no other detailed studies on the economic impact of sharing parental material in Germany and so far no other paper has quantitatively analyzed the impact of sharing genetic material on the overall production of winter wheat in Germany or any other region. Byerlee and Dubin (2009) note that that there are no quantitative evaluations on how plant variety rights and patenting affect germplasm flows in the breeding cycle. This paper contributes to filling this research gap. With our unique data set we can comprehend the inflow of external parental material to winter wheat breeding programs and hence provide a first evaluation of the impact of the breeder’s exemption on winter wheat production in Germany. Most papers within this thematic area concentrate on the loss of plant genetic resources for agriculture and limits to their access as well as international collaboration to exchange these (see e.g., Esquinas-Alcázar, 2005; Rubenstein et al., 2005; McCouch, 2013; Ogwu et al., 2014). Furthermore, most of these studies concentrate on low- and middle-income countries, whereas our study explores a high-income country. In the field of agricultural sciences, the breeder’s exemption is not a research focus. Outside the field, the exemption and its innovation and competition character have been more often analyzed from disciplines such as law (e.g., Heald and Chapman, 2012), sociology (e.g., Brandl, 2018) as well as general economics (e.g., Koo et al., 2006).

Some studies also estimate the impact of wheat breeding research on different agricultural economic indicators such as monetized yield increases or forgone yield decreases as well as price changes due to supply changes (see Byerlee and Traxler, 1995; Heisey et al., 2002; Evenson and Rosegrant, 2003; Marasas et al., 2004; Byerlee and Dubin, 2009; Lantican et al., 2016). Our economic surplus estimates are lower than those estimates of the listed studies, as they calculated the impact of overall breeding, not only the exchange of parental material. Further, they focused on developing countries in the global South, whereas this study is limited to German winter wheat breeding. In Germany, systematic wheat breeding and production with improved varieties has been present since centuries. Moreover, the above-mentioned studies focus on other wheat strains than winter wheats. Additionally, in contrast to existing studies, we did not include the effect’s repercussions on other breeding systems in similar agricultural environments due to the scope of our study and the available data.

Given the rapid changes and high interannual fluctuations in agronomic conditions due to aggravating climate change, one could suggest that breeders who have access to a broader gene pool can create better-adapted varieties as they have a larger working basis. The use of external material offers the opportunity to use more variable genetic resources that have important traits for climate change adaptation (Searchinger et al., 2014). To advance the progress of yield and other traits, breeders can further exploit the large pool of genetic resources of released varieties, landraces and wild relatives, if they have access to it (Fischer et al., 2014). Sharing genetic material helped yield progress, but it has become more difficult due to barriers by international treaties, governments without full information, and interests from the private sector (Fischer et al., 2014).

We estimate the benefits of exchanging genetic resources by the yield gain that is attributable to wheat breeding (see Voss-Fels et al., 2019). We must reiterate that the presented results are derived by an *ex post* assessment. Because of this, market shifts, that might have occurred if the restrictions laid out in the scenarios had been implemented in the 1970s, are not included. Based on our scenarios, farmers could have only selected and planted 0E-varieties. Hence, breeders would have adjusted their breeding approaches. In those years when our data set shows a zero production of 0E-varieties, as no such varieties were multiplied, farmers could have planted a (second-best) winter wheat 0E-variety or switched to another crop or land use. The variety-specific multiplication area shows that farmers have only limitedly planted 0E-varieties despite their availability and higher yields in some years.

Further research in this area should enhance and increase the data base regarding the exchange of genetic material in German winter wheat breeding. To prevent the problem of incomplete pedigrees, future work could use other proxies for the breeder’s exemption or the exchange of genetic resources. Moreover, focus groups with breeders could provide additional insights in the way the breeders use the exemption and into the questions of which practical hurdles breeders must overcome to exchange material.

## Conclusion

The results of this assessment show that the use of other breeders’ varieties, enabled by the breeder’s exemption, was standard in German winter wheat breeding for those varieties that were planted between 1972 and 2018. This shows that the exchange is a crucial part of the work carried out by breeders. The breeder’s exemption ensures that breeders can access a large pool of genetic material and create a larger effective population size as a basis for their breeding activities. While varieties that were created without exchange had a superior unweighted yield, those varieties created with exchange had an advantage when weighing the yield by farmers’ adoption. This discrepancy between weighted and unweighted grain yield shows that farmers’ preferences are based on multiple factors. Thereby yield is one important factor, but also resistances and tolerances to abiotic and biotic stresses and for example farmers’ previous experiences play a crucial role. We concentrated on grain yield as it can be seen as a general performance indicator that also mirrors other traits and hence is also important for breeders.

The extent of adoption was measured by the variety-specific multiplication area. The yearly average of the multiplication area of those varieties with two external parents was seven times as big, the area of those varieties with one external parent four times as big as the area of those varieties with no external parents. The higher adoption-rate translated into higher group production: group 2E contributed five times, and group 1E three times more to overall German winter wheat production. From an economic perspective the breeder’s exemption has zero costs, nevertheless it generated a positive economic surplus. Translating these differences in farmers’ adoption or market penetration into monetary terms, an economic surplus of 19.2 to 22.0 billion Euros was generated from 1972 to 2018 by exchanging parental breeding material.

These insights can also guide decision making in other institutional or agro-climatic environments, as we investigated the impact of exchanging genetic resources in a system where breeders have the freedom of choice to access a large genetic gene pool. The necessity and use of the breeder’s exemption are debated in Europe, in particular in comparison to patent-systems. To the best of our knowledge, the benefit of exchanging breeding material in German winter wheat breeding has not been quantified by agricultural economists. Hence, our work, that analyzes the most grown crop in Germany, can provide a data-based insight to this debate. We performed a pedigree analysis for German winter wheat varieties and set up scenarios to quantify if a system with limited exchange would have generated other production quantities. Agricultural production is under pressure from multiple sides. Therefore, it needs to become more sustainable with less impacts on the environment and climate. Our research has shown that market penetration was higher for those varieties with external parents. This shows that breeders’ access to a wide gene pool is an important prerequisite for a sustainable agriculture that feeds the world under climate change in the future.

## Data Availability Statement

The raw data supporting the conclusions of this article will be made available by the authors, without undue reservation.

## Author Contributions

SL, BW, CG, and SN designed the research. SL performed the research and wrote the manuscript with contributions from BW, CG, and SN. SN initiated the research. HL-C reviewed the economic surplus approach and contributed to the interpretation of the results. All authors contributed to the article and approved the submitted version.

## Conflict of Interest

SL and SN are employed by the company HFFA Research GmbH. The work was carried out within the project BRIWECS, funded by the German Federal Ministry of Education and Research (BMBF Grant No: 031A354E). The remaining authors declare that the research was conducted in the absence of any commercial or financial relationships that could be construed as a potential conflict of interest.

## Figures and Tables

**FIGURE 1:**
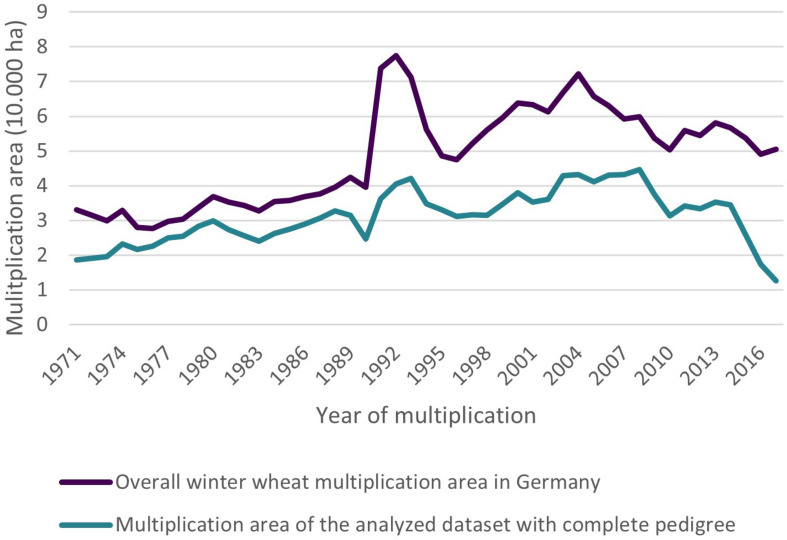
Development of the overall winter wheat multiplication area from 1971 to 2017 in Germany and the area of all varieties in the analyzed data set with full pedigree information, Source: Own calculations with data from Kempf (2019).

## Footnotes

1 http://www.wheatpedigree.net/

## Annex I

**TABLE 1:** Pairwise comparisons using Wilcoxon rank sum test for the variable grain yield (*p*-values).

Group	2E	1E	E
1E	3.90E-07		
0E	9.90E-12	2.10E-04	1.50E-09

**TABLE 2:** Pairwise comparisons using Wilcoxon rank sum test for the variable multiplication area (*p*-values).

	2E	1E	E
1E	< 2E-16		
0E	1.30E-01	< 2E-16	1.60E-05

**TABLE 3:** Pairwise comparisons using Wilcoxon rank sum test for the variable weighted yield (*p*-values).

	2E	1E	E
1E	3.70E-03		
0E	6.08E-02	9.88E-01	1.55E-01

**TABLE 4:** Pairwise comparisons using Wilcoxon rank sum test for the variable group production (*p*-values).

	2E	1E	E
1E	3.30E-05		
0E	2.00E-15	3.30E-05	3.90E-16

## Annex II

**TABLE 5:** Scenario-induced production shifts and economic surplus over time.

YEAR OF PRODUCTION	INDUCED PRODUCTION SHIFT (MILLION T)		ECONOMIC SURPLUS (MILLION EUR)	
	Scenario I	Scenario II	Scenario I	Scenario II
1972	2.8	5.9	491.8	1041.6
1973	3.7	6.2	667.8	1128.2
1974	0.2	0.2	36.1	34.7
1975	4.1	5.6	853.4	1178.1
1976	0.2	0.2	48.7	52.6
1977	0.2	0.2	54.0	55.8
1978	0.3	0.3	64.7	65.1
1979	0.3	0.3	74.9	73.2
1980	0.4	0.4	91.8	90.8
1981	0.5	0.5	107.9	106.7
1982	0.4	0.4	95.6	93.7
1983	0.5	0.5	119.4	118.3
1984	9.3	9.8	2123.8	2246.2
1985	8.6	9.4	1774.5	1938.6
1986	8.4	10.0	1727.1	2047.7
1987	8.0	9.6	1609.8	1916.7
1988	10.2	11.5	1874.1	2122.0
1989	9.8	10.7	1733.9	1903.9
1990	13.6	15.0	2300.7	2531.8
1991	14.5	16.2	2423.7	2701.3
1992	−0.4	−0.5	−58.0	−84.9
1993	0.1	−0.1	9.7	−11.4
1994	0.2	0.0	23.3	−1.8
1995	0.5	0.3	63.3	43.0
1996	0.4	0.3	57.0	37.3
1997	0.5	0.5	57.7	55.3
1998	1.0	1.5	106.3	169.0
1999	0.4	0.7	49.3	73.8
2000	0.3	0.3	29.3	33.5
2001	0.4	0.1	38.8	7.8
2002	0.2	−0.1	16.4	−10.3
2003	−0.1	−0.4	−10.5	−39.1
2004	−0.1	−0.4	−15.8	−46.1
2005	−0.1	−0.3	−7.6	−27.0
2006	−0.1	−0.3	−9.8	−33.1
2007	0.0	−0.2	−7.0	−36.1
2008	0.0	−0.2	−3.0	−31.9
2009	0.0	−0.1	3.2	−10.0
2010	0.1	0.0	13.4	−6.0
2011	0.1	−0.1	22.1	−20.5
2012	0.2	0.0	49.4	6.5
2013	0.4	0.2	75.7	38.9
2014	0.6	0.5	97.7	76.9
2015	0.5	0.5	81.9	70.0
2016	0.5	0.4	69.0	60.7
2017	0.5	0.4	78.6	64.2
2018	0.7	0.6	112.8	99.9
SUM	**102.6**	**116.3**	**19,217.1**	**21,925.3**
AVERAGE	**2.2**	**2.5**	**408.9**	**466.5**
